# *Mycobacterium tuberculosis* virulence inhibitors discovered by *Mycobacterium marinum* high-throughput screening

**DOI:** 10.1038/s41598-018-37176-4

**Published:** 2019-01-10

**Authors:** Hasan Tükenmez, Isabel Edström, Ramesh Ummanni, Stina Berglund Fick, Charlotta Sundin, Mikael Elofsson, Christer Larsson

**Affiliations:** 1 Infectious Diseases Clinic, Umeå University Hospital, 901 85 Umeå, Sweden; 2 Department of Molecular Biology, Umeå University, 901 87 Umeå, Sweden; 3 Department of Applied Biology, CSIR-Indian Institute of Chemical Technology (CSIR-IICT), Tarnaka, Hyderabad, 500007 Telangana India; 4 Department of Chemistry, Umeå University, 901 87 Umeå, Sweden

## Abstract

High-throughput screening facilities do not generally support biosafety level 3 organisms such as *Mycobacterium tuberculosis*. To discover not only antibacterials, but also virulence inhibitors with either bacterial or host cell targets, an assay monitoring lung fibroblast survival upon infection was developed and optimized for 384-plate format and robotic liquid handling. By using *Mycobacterium marinum* as surrogate organism, 28,000 compounds were screened at biosafety level 2 classification, resulting in 49 primary hits. Exclusion of substances with unfavourable properties and known antimicrobials resulted in 11 validated hits of which 7 had virulence inhibiting properties and one had bactericidal effect also in wild type *Mycobacterium tuberculosis*. This strategy to discover virulence inhibitors using a model organism in high-throughput screening can be a valuable tool for other researchers working on drug discovery against tuberculosis and other biosafety level 3 infectious agents.

## Introduction

Drug resistant tuberculosis (TB) is a major health threat and the World Health Organization (WHO) estimates that 490,000 new cases of multidrug-resistant TB (MDR-TB) occurred in 2016^1^. Use of second line drugs extends the duration of treatment from 6 months to 12 or more and reduces the success rate substantially. Only 54% of MDR-TB and 30% of extensively drug-resistant TB (XDR-TB) are successfully treated^1^. Today it is challenging, sometimes even impossible to find a treatment regimen that is both effective against the bacteria and tolerated by the patient. Despite the obvious need for new antibiotics, only two new TB drugs, bedaquiline and delamanid, have been developed over the last 40 years. Bedaquiline was approved by the Food and Drug Administration (FDA) in 2012 and approved by the European Medicines Agency (EMA) 2014 but already at the end of that year the first case with a resistant strain was reported^2^, and there are today several case reports of TB resistant to both bedaquiline and delamanid^3,4^. Due to the increasing problem with drug resistant *M. tuberculosis* there is a desperate need for new drugs with new mechanisms of action.

High-throughput screening (HTS) is a commonly used approach to screen large numbers of synthetic or natural compounds for antimicrobial properties. Most HTS assays are designed to find compounds killing the microbe or inhibiting its multiplication. These type of screens have generated compounds such as bedaquiline^5^, and 6-aryl-5,7-dimethyl-4-phenylcoumarin compounds inhibiting mycobacterial FadD3 2acyl–acyl carrier protein synthase activity in murine TB^6^. The vast majority of compounds available in commercial libraries have already been screened for antimycobacterial activity and very few new compounds are therefore expected to be discovered. Since HTS is highly dependent on advanced liquid handling robots and analytical instruments, it is generally inaccessible to drug discovery endeavours using wild type (WT) *M. tuberculosis* or other biosafety level 3 (BSL3) pathogens. Only few institutions are capable of performing HTS under BSL3 conditions and time on the instruments is then regularly scarce and costly. This constitutes a major bottleneck in the search for new drugs against TB in general and in discovery of virulence inhibitors in particular since attenuated strains such as *M. bovis* Bacille Calmette-Guerin (BCG) or *M. tuberculosis* H37Ra is then of limited use.

Our work aiming to discover virulence inhibitors against TB was inspired by the BCG vaccine used in TB prevention. BCG is a *M. bovis*, closely related to *M. tuberculosis*, rendered avirulent due to repeated passaging *in vitro*^7^. The genetic origin of attenuation is mainly the loss of three regions, regions of difference (RD) 1-3^8–10^. The RD-1 contains the important ESX-1 secretion system and proteins such as ESAT-6 and Cfp-10 important for bacterial virulence but not growth^11,12^. Bacterial virulence mechanisms are attractive targets for drug development since they generally are specific for a certain pathogen and thereby not affecting commensal microorganisms. They may also be less selective for development of drug resistance since bacterial division is not targeted. Parts of our team have previously discovered and further developed virulence inhibitors of type III secretion in *Yersinia pseudotuberculosis* and *Chlamydia trachomatis*^13–15^. With this foundation, we have performed a HTS endeavour to discover mycobacterial virulence inhibitors.

To circumvent the need of a BSL3 screening facility, we have developed a HTS strategy to discover mycobacterial virulence inhibitors using the fish and frog pathogen *Mycobacterium marinum* as a model organism for *M. tuberculosis. M. marinum* shares 3,000 homologous genes with *M. tuberculosis* with an average amino acid identity of 85% and is fully virulent in cold-blooded animals. Moreover, *M. marinum* is a BSL2 organism that can be utilized in regular HTS facilities. We screened 28,000 chemical compounds with *M. marinum* under BSL2 conditions and discovered 11 novel hits as potential mycobacterial virulence inhibitors. The hits were further tested with fully virulent *M. tuberculosis* under BSL3 conditions and eight of these had similar virulence inhibiting properties in both strains. The HTS strategy developed in this study can be a valuable tool in TB drug discovery, as it provides the opportunity to screen for virulence inhibitors under BSL2 conditions.

## Results

### High-throughput screening in *M. marinum*

**Figure 1:**
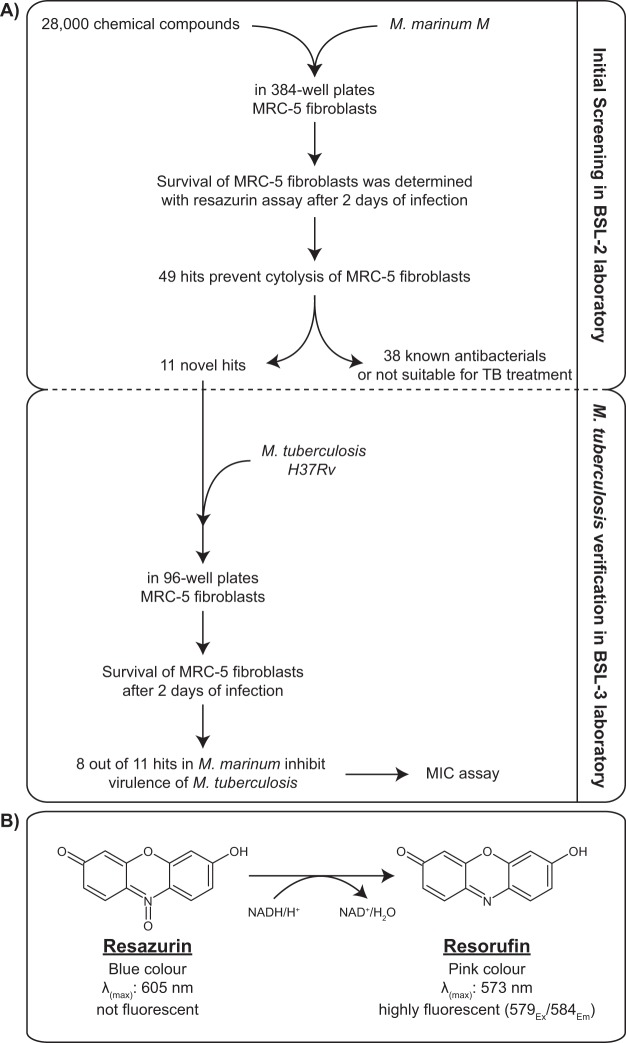
High-throughput screening assay and validation strategy. (**A)** The discovery phase including high-throughput screening, initial hit validation and dose response experiments performed with *M. marinum* in 384-well format using HTS robotics. In the validation phase, effect of the hit compounds was verified with *M. tuberculosis* with further dose response experiments and antimicrobial activity of selected hit compounds were performed manually in 96-well format in a BSL3 laboratory. (**B**) Reduction of resazurin to resorufin as response to respiration.

To develop a HTS method for discovery of not only antimicrobials, but any type of compound having an adverse effect on mycobacterial virulence, we took advantage of the cytolytic properties also present in *M. marinum*. Phylogenetically, *M. tuberculosis* is considered a “downsized version of *M. marinum”*^16^. Using *M. marinum*, we performed a primary screening of 28,000 small molecules from a diverse set of compounds (ChemBridge and CBCS primary screening set) in 384-well format at our BSL2 level screening platform. Our assay strategy is based on survival of MRC-5 human lung fibroblast cells upon infection with *M. marinum* in the presence of various compounds at a concentration of 20 μM for 48 hours. After compound exposure, all wells were washed in order to remove bacteria and detached dead MRC-5 cells. Activity of the remaining MRC-5 cells was determined by a resazurin conversion assay (see materials and methods). With this strategy, virulence inhibitors with bacterial targets, host-directed immunomodulatory compounds as well as classical antibiotics with bacteriostatic or bactericidal activity can be discovered **(**Fig. 1**)**.

Cell density, multiplicity of infection (MOI), incubation time, temperature and medium composition were all adjusted to maximize the window of discovery between the WT and the attenuated control strain lacking the RD1 region (*∆RD1*). These parameters were also optimized not to kill off all MRC-5 cells by the WT strain to minimize the risk of missing active substances. It can be noted that the serum concentration in the A-DMEM cell culture medium is a crucial factor since too high concentration allow for the MRC-5 cells to overgrow the effect from bacteria and too low concentration cause detachment from the microtiter well bottom and poor viability. Reduced serum in infection models have been used previously by e.g. Rastogi *et al*. and Mehta *et al*.^17,18^. Despite the challenging screening assay, with many steps and additions of cells and bacteria, as well as long incubation time and washing of wells prior to detection of MRC-5 cell viability, we were able to generate an assay with average Z-values of around 0.3 that is considered as a doable assay^19^, in particular for being a phenotypic assay. Out of 28,000 compounds, 267 primary hits were selected, defined by increasing the viability of MRC-5 by ≥3 standard deviations from the negative control (DMSO).

All selected hits were re-tested in duplicates resulting in 49 confirmed hits that were subjected to full dose-response analysis in *M. marinum* (data not shown). Of the 40 substances showing favourable dose response curves, 29 compounds were excluded as known antibiotics, such as linezolid, moxifloxacin and several members of the rifampicin family, or due to undesirable properties such as being known immunosuppressants, having anesthetic properties or being commonly encountered hits such as chelating agents or absorbents of ultraviolet light. This process resulted in 11 compounds selected for further evaluation. Compound structures and physicochemical properties calculated by QikProp 5.2 and Maestro 11.2 software (Schrödinger Inc.) are provided in Supplementary Table 1 and Fig. 1.

### Hit verification in *M. tuberculosis*

The remaining 11 hits were subsequently tested with fully virulent *M. tuberculosis* H37Rv in 96-well format under BSL3 conditions. The MRC-5 survival assay was slightly adjusted to *M. tuberculosis* and BSL3 conditions (see materials and methods). The attenuated BSL2 version of this strain, H37Ra, also lacking the RD1 region was used as positive control analogous to *M. marinum ∆RD1*. We then evaluated selected compounds at various concentration from 2.5 to 40 μM.

**Figure 2:**
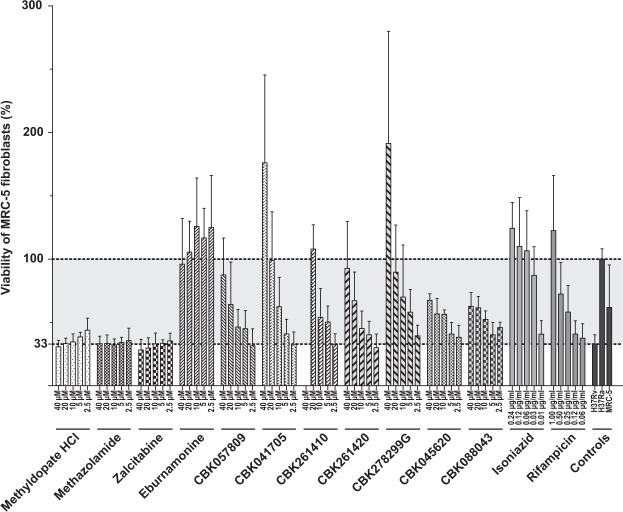
Survival of MRC-5 fibroblasts after two days of infection with *M. tuberculosis* in the presence of various concentrations of hit compounds. The viability of MRC-5 fibroblasts was determined by resazurin conversion assay (see materials and methods). The viability of MRC-5 fibroblasts that were exposed to avirulent *M. tuberculosis* H37Ra was set to 100% (upper dotted line) and the rest of the samples were normalized accordingly. In the absence of any compounds, exposure to virulent H37Rv results in 33% viability compared to H37Ra exposure (lower dotted line). In addition to the hit compounds, first-line antibiotics isoniazid and rifampicin were also included as controls. The bar graph was plotted based on average and standard deviation values obtained from at least four independent replicates.

Of these 11 hits, methyldopate HCl, methazolamide and zalcitabine substances showed no activity in *M. tuberculosis* at 40 μM which is twice the screening concentration **(**Fig. 2**)**. To evaluate the screening strategy and the differences between *M. marinum* and *M. tuberculosis* drug discovery, we tested the compounds inactive at 40 μM at higher concentrations. Methyldopate HCl is an inhibitor of the enzyme DOPA decarboxylase used in treatment of hypertension. Although showing promise in *M. marinum*, the compound has no effect in the *M. tuberculosis* infection apart from a negligible increase in resazurin conversion at 160 µM and toxicity to the MRC-5 cells at 320 µM **(**Supplementary Fig. 2**)**. Methazolamine is a carbonic anhydrase inhibitor used to treat ocular hypertension. In our assay, it has an effect close to H37Ra levels at 320 µM which is lower at 160 µM and nonexistent at 80 µM or lower concentrations **(**Supplementary Fig. 2**)**. The extreme concentrations needed for protection makes it less attractive as a starting point for drug development. Also, zalcitabine or 2′-3′-dideoxycytidine, a pyrimidine analog previously used for treatment of human immunodeficiency virus/acquired immunodeficiency syndrome (HIV/AIDS) had activity against *M. marinum* but not to *M. tuberculosis* even at a concentration of 320 µM **(**Supplementary Fig. 2**)**.

**Figure 3:**
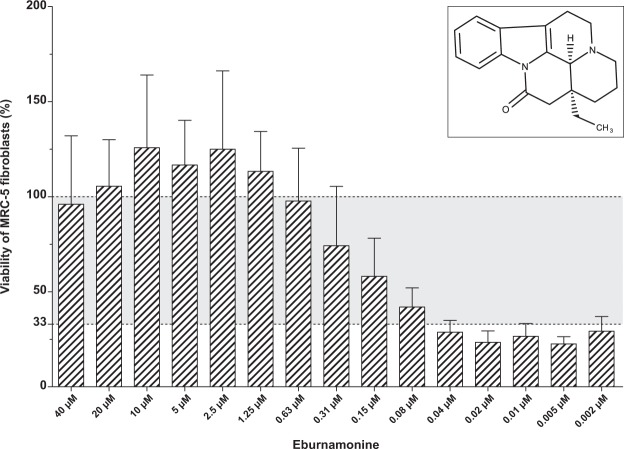
Survival of MRC-5 fibroblasts after two days of infection with *M. tuberculosis* in the presence of various concentrations of eburnamonine. The viability of MRC-5 fibroblasts was determined by resazurin conversion assay (see materials and methods). The viability of MRC-5 fibroblasts that were exposed to avirulent *M. tuberculosis* H37Ra was set to 100% (upper dotted line) and the rest of the samples were normalized accordingly. In the absence of any compounds, exposure to virulent H37Rv results in 33% viability compared to H37Ra exposure (lower dotted line). The bar graph was plotted based on average and standard deviation values obtained from at least four independent replicates.

The most potent compound among the hits was the indole alkaloid eburnamonine, a natural product from the Madagascar periwinkle (*Catharanthus roseus*). Eburnamonine is a vasodilatory compound also shown to have cytotoxic effect on leukemic lymphocytes^20,21^. In our assay with *M. tuberculosis* infecting MRC-5 cells, the cells show a resazurin conversion similar to cells exposed to avirulent H37Ra at an eburnamonine concentration as low as 0.63 µM with a dose-dependent decline in effect noticeable down to 0.08 µM **(**Fig. 3**)**. Both CBK057809 and CBK261420 restore cell viability to about 90% of H37Ra infection at 40 µM. At 20 µM viability is about 65% and decreasing with lower concentration **(**Fig. 2**)**. CBK041705 displays good effect at 20 µM with a concentration dependent decrease in protection at lower concentrations. CBK261410 has 100% activity compared to H37Ra at 20 µM but is cytotoxic at 40 µM **(**Fig. 2**)**. The only antibacterial compound was CBK278299G with bactericidal effect after 13 days at 40 µM and noticeable reduced growth at 20 µM assayed by pin spotting on nonselective 7H10 agar and pellet formation in U-bottom culture plate **(**Supplementary Figs 3 and 4**)**. CBK045620 and CBK088043 had a moderate protective effect of about 60% of H37Ra at 20 µM with negligible improvement at 40 µM **(**Fig. 2**)**.

### Effect of hit compounds on macrophage-like cell infection

**Figure 4:**
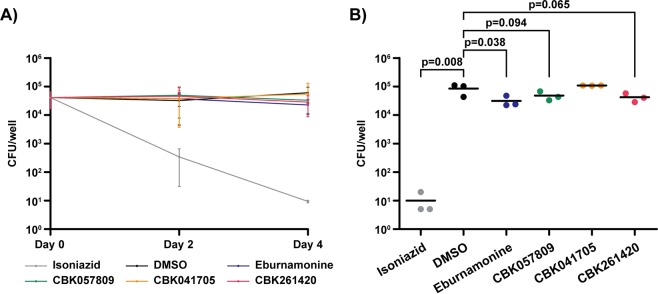
Intracellular survival of *M. tuberculosis* in J774A.1 macrophage-like cells exposed to hit compounds. (**A**) Intracellular bacteria in J774A.1 cells exposed to 40 µM of hit substances at day 0, 2 and 4 of infection. 0.4% DMSO and 0.24 μg/ml isoniazid were included as negative and positive controls. (**B**) Scatter dot plot of bacterial numbers on day four of infection. Graphs are plotted as average and standard deviation obtained from three independent replicates. Significance levels are calculated using the one-tailed student’s t-test.

In this project, the main focus has been discovery of substances capable of preventing fibroblast lysis. In order to study the effect of discovered hit compounds in a biologically more relevant intracellular infection model, we infected the macrophage-like cell line J774A.1 with 10 MOIs of *M. tuberculosis*, washed away extracellular bacteria and exposed the cells to 40 µM of hit compounds. We monitored bacterial numbers at days zero, two and four of infection by lysing the cells and plate intracellular bacteria on 7H10 plates for viable count **(**Fig. 4**)**. On day four, DMSO-treated control wells had a slight increase in bacterial numbers compared to day zero, whereas isoniazid treated cells displayed a four log drop. Of the experimental compounds, eburnamonine exposure resulted in a small but statistically significant (*p* = 0.038) 3-fold reduction in bacterial numbers whereas the 2-fold reductions of bacteria in cells treated with CBK261420 (*p* = 0.065) and CBK057809 (*p* = 0.094) were not significant within a 5% cutoff **(**Fig. 4B**)**. Viability of J774A.1 cells infected with 10 MOI *M. tuberculosis* and exposed to 40 µM hit substances was assessed with the resazurin assay on day 4 as described above. No cytotoxicity was observed either from infection or compound exposure (data not shown).

## Discussion

In this project, we set out to discover molecules that inhibit virulence rather than target bacterial division or survival as conventional antibiotics do. The BCG vaccine strain lacking critical virulence factors has been a major inspiration and our goal is to achieve a similar pacifying effect by a small organic molecule. Instead of searching for compounds inhibiting growth, we screened for compounds capable of rescuing host cells from lysis. In the primary screen, we used the human lung fibroblast cell line MRC-5 which is lysed by mycobacteria through secretion of the cytolytic protein ESAT-6 **(**Fig. 1**)**. Since *M. tuberculosis* requires a BSL3 laboratory, we chose to use the related bacterium *M. marinum*, a pathogen of cold-blooded aquatic animals such as fish and frogs to screen using the technical facilities at our HTS platform.

Our strategy is based on screening compounds for their capability to rescue MRC-5 cells upon infection with *M. marinum*. Therefore, this approach has potential to discover virulence inhibitors and molecules with protecting properties that target the host cells, also known as immunomodulators or “host directed therapy”, in addition to conventional antibiotics. In a similar, coinciding screening project using *M. tuberculosis* and MRC-5 cells under BSL3 conditions, Rybniker and co-workers discovered benzyloxybenzylidene-hydrazine and benzothiophene both having an impact on ESX-1 secretion^22^. This is an elegant demonstration of the potential of cell-based infection assays to discover virulence inhibitors. Moreover, in this type of cell survival assays, compounds with general toxicity to eukaryotic cells are to a large extent excluded already in the primary screen. To minimize the risk of missing active compounds, we designed the assay to not kill off all MRC-5 cells by H37Rv. In our controls exposed to the avirulent H37Ra, we consistently observed a higher resazurin conversion compared to uninfected cells and wells with H37Rv and active experimental compounds often displayed even higher resazurin conversion **(**Fig. 2**)**. In an excellent review on metabolic host responses to infection by intracellular bacterial pathogens, Eisenreich *et al*. describe a set of general “core host responses” to bacteria in general, even avirulent and heat-killed^23^. One of these are NF-κB induced by pathogen-associated molecular patterns, regulating energy homeostasis and metabolic adaptations by upregulating mitochondrial respiration^23,24^. We hypothesize the higher resazurin conversion in H37Ra exposed cells compare to uninfected control is due to increased MRC-5 metabolism in response to bacterial contact.

Among the 40 primary screening hits showing favourable dose response curves, 19 were well known antibiotics active against TB, demonstrating the power of the screening strategy in discovering active compounds. The MIC assay performed with *M. tuberculosis* revealed that among the 11 re-evaluated hits with *M. tuberculosis* **(**Supplementary Fig. 1**)**, only CBK278299G resulted in reduced bacterial growth at 40 µM **(**Supplementary Fig. 2**)**. Moreover, failure of recovery when spotted on 7H10 agar plates without antibiotics indicates bactericidal activity **(**Supplementary Fig. 2**)**.

We do observe some discrepancies between the primary screen in *M. marinum* and the *M. tuberculosis* verification experiments as 2 out of 11 *M. marinum* hits have no activity in *M. tuberculosis* even at concentrations as high as 320 µM and one compound shows activity only at concentrations 160–320 µM. This difference in susceptibility between two distinct but similar species is not very surprising keeping in mind the pyrazinamide resistance of *M. tuberculosis* complex bacterium *M. bovis* due to a single H57D substitution in *pncA* and even the importance of *M. tuberculosis* strain choice in *in vivo* drug testing^25,26^.

Lung fibroblasts are not major host cells in *M. tuberculosis* infection and in the assay presented here they are used solely for measuring cytolytic activity. In order to assess the effect of hit compounds in a biologically relevant, phagocytic host cell, we used the macrophage-like J774A.1 originally derived from a BALB/c mouse tumour and infected it with *M. tuberculosis* at 10 MOI **(**Fig. 4**)**. At day four post infection, cells treated with 40 µM eburnamonine contained 1/3 of the bacteria in DMSO controls (*p* = 0.038). Although statistically significant, this is far from the four-log reduction resulting from exposure to 0.24 μg/ml isoniazid, an antibiotic that is bactericidal to dividing mycobacteria, and bacteriostatic to slow-growing. CBK261420 and CBK057809 showed a non-significant (*p* = 0.065 and *p* = 0.094 respectively) 2-fold reduction compared to the DMSO control and CBK057809 which harboured as many bacteria. In contrast to isoniazid, the hit compounds discovered here show neither bactericidal nor bacteriostatic properties with the exception of CBK278299G which displayed a weak bactericidal effect at 40 µM **(**Supplementary Fig. 2**)**. These results, with only minor reduction **(**Fig. 4**)**, if any, in bacterial numbers are what we expect from substances with virulence inhibition or host response modulating properties with no effect on bacterial growth within the time frame of an *in vitro* experiment. Upcoming research will focus on evaluating the benefit of hit compounds in animal models in combination with the standard treatment regimen, with particular focus on reduction of pathology and host cell death, but also bacterial loads in a time frame of weeks to months.

In summary, eburnamonine followed by CBK057809, CBK041705, CBK261410 and CBK261420 are the most promising hits with virulence inhibiting properties *in vitro* **(**Fig. 2**)**. CBK057809, a *N,N’* disubstituted urea compound, and the 1,1-diphenylethyl compound CBK041705 are relatively small compared to CBK261410 and CBK261420 that both are large lipophilic compounds containing multiple aromatic rings. Both the later compounds contain a stereocenter and was screened and evaluated as their respective racemate. This opens up the possibility to explore stereochemistry in future medicinal chemistry programs. None of these compounds have, to the best of our knowledge, previously been shown to possess virulence blocking properties against mycobacteria. Further research in expanding the scaffolds, evaluating them in other assays, discover their targets and impact on bacterial or host cell physiology is ongoing. These compounds can be employed as starting points in development of virulence inhibiting drugs against TB. The results also show that our strategy of screening in a BSL2 model organism is a valid approach to identify virulence inhibitors against the BSL3 pathogen *M. tuberculosis*.

## Materials and Methods

### Cell culture

Human lung fibroblast cell line MRC-5 (ATCC^®^ CCL-171™) and macrophage-like cell J774A.1 derived from BALB/c mouse tumour (ATCC^®^ TIB-67^™^) were maintained and expanded in Advanced DMEM (A-DMEM) (Life technologies) supplemented with 5% new-born calf serum and 0.3% (w/v) L-glutamine at 37 °C with 5% CO_2_ until 80% confluent.

WT *M. marinum* M (ATCC BAA-535™) and a *RD1* knockout with *M. marinum* M background (a kind gift from Fredric Carlsson, Lund university) were grown in 7H9 medium (BD Difco) supplemented with 0.05% Tween80 and 10% ADS (0.5% albumin, 0.2% dextrose and 0.085% saline) as standing cultures at 30 °C. *M. tuberculosis* H37Rv and H37Ra were also grown in 7H9 medium supplemented with 0.1% Tween80 and ADS as standing cultures at 37 °C.

### High-throughput screening

MRC-5 cells were detached from culture flasks by adding 0.25% (w/v) Trypsin - 0.53 mM EDTA and suspended in A-DMEM with 3% FBS. Cells were counted in a Countess™ automated cell counter (Invitrogen) and concentration adjusted to 1.2 × 10^5^ cells/ml in A-DMEM with 3% FBS. A Multidrop dispenser (MTX lab systems) was used to seed 25 µl cell suspension to clear, flat bottomed 384 well plates (NUNC) resulting in 3 × 10^3^ cells/well.

After 6 hours of incubation at 37 °C to let cells attach to the wells, 5 μl experimental compounds in serum-free S-MEM (Life Technologies) was added to the screening plate using a liquid handling robot (Biomek NX, Beckman Coulter) to obtain a final concentration of 20 μM.

Mycobacterial inoculum was prepared by pelleting bacteria by centrifugation at 14,000 g for 3 minutes. The pellet was washed twice in HBSS (Life technologies), resuspended in serum-free S-MEM and passed through a 27 G x ¾” needle (BD Microlance™) to break up bacterial aggregates into single-cell suspension. Optical density (OD_600_) was adjusted to 0.096 and 20 μl was added to screening plates using a Multidrop dispenser, resulting in a multiplicity of infection (MOI) of 20 bacteria/cell. S-MEM only was added to negative control wells and the isogenic, RD1 deficient strain *M. marinum ΔRD1* served as positive control. Each plate had positive controls (*∆RD1*), negative controls (WT *M. marinum*) as well as MRC-5-cells-only and medium-only controls, all with same DMSO concentration as compound wells. Screening plates were sealed with adhesive film and incubated at 37 °C for 48 hours.

Plates were washed using the Biomek liquid handling robot with following settings: 40 μl media was removed from each well, leaving 10 μl/well. 60 μl PBS (pre-warmed to 37 °C) was added and liquid was pipetted up and down three times before 60 μl was aspirated and discarded. Tips were washed in sterile water followed by disinfection in 70% ethanol and a wash step in another plate with sterile water before continuing with the next assay plate.

To quantify cellular survival, 40 μl 50 mM resazurin (Sigma-Aldrich) in 1:1 S-MEM:A-DMEM with 3% FBS was added to wells and plates were incubated at 37 °C with 5% CO_2_ for 2.5 hours or until the positive control wells turned pink. Conversion from resazurin to resorufin was then determined by fluorescence measurement of resorufin on a TECAN plate reader at excitation 560 nm and emission 590 nm.

The LCBU primary screening set (~17,500 compounds, ChemBridge) and ~7,300 compounds of the CBCS primary screening set (Chemical Biology Consortium Sweden) were screened at 20 µM concentration as singlets. Positive hits were selected using MScreen (University of Michigan) as ≥3 standard deviations above the DMSO control^27^. All selected hits were ECHO spotted (Labcyte) and tested in the same screening setup as in the primary screen, but in duplicates.

For dose-response assessments, compounds were spotted in triplicates in 7 different dilutions from 100 µM to 1.56 µM and analysed using the same experimental strategy as aforementioned HTS.

### Activity in *M. tuberculosis*

To evaluate screening hits obtained in *M. marinum*, the assay was slightly modified and optimised for *M. tuberculosis* and BSL3 facilities. The assay protocol was partly altered to function in *M. tuberculosis* and BSL3 conditions. MRC-5 cells were counted using a Neubauer improved, 0.1 mm depth counting chamber (Brand^®^) and concentration adjusted to 4 × 10^5^ cells/ml in A-DMEM with 3% FBS. 50 μl cell suspension was then manually added to clear, flat bottomed 96 well plates (Thermo Scientific) using an 8-channel multipipette resulting in 2 × 10^4^ cells/well.

After 6 hours of incubation at 37 °C to let cells adhere to the wells, 25 μl compound solution in serum-free S-MEM was manually added to the wells. For dose-response assessments, the final compound concentration tested varied from 320 μM to 0.002 μM. Isoniazid and rifampicin at varying concentrations were also used as additional controls.

*M. tuberculosis* H37Rv and its attenuated derivative H37Ra were pelleted by centrifugation at 15,000 g for 3 minutes, washed once with serum-free S-MEM and resuspended in serum-free S-MEM. Bacterial suspension was passed through a 27 G x ¾” needle (BD Microlance™) to break up bacterial aggregates into single-cell suspension. Optical density (OD_600_) was adjusted to 0.76 and 25 μl was manually added to the assay plates, resulting in MOI of 30 bacteria/cell. Each assay plate had H37Ra as positive control, H37Rv with DMSO instead of compounds as negative control, as well as MRC-5 cells only and medium only controls. Assay plates were incubated at 37 °C with 5% CO_2_ for 48 hours with regular plate lid.

After all media in the wells had been manually removed using an 8-channel pipette, wells were washed with 100 μl 37 °C PBS. This was then followed by addition of 100 μl 100 μM resazurin in A-DMEM with 5% FBS and plates were incubated at 37 °C with 5% CO_2_ for 4–5 hours. Levels of resazurin and resorufin were determined by absorbance measurement at 595 nm (resazurin) and 570 nm (resorufin) on EMax plus plate reader. The ratio between these optical densities (A_570_/A_595_) were then used to determine the conversion rate from resazurin to resorufin.

For each assay plate, A_570_/A_595_ ratios were calculated for each well. Average value obtained from medium-only controls was subtracted. Finally, average value obtained from wells containing H37Ra positive controls were set to 100% indicating total MRC-5 cell survival and the values from other wells were normalized according to this positive control. Each compound’s impact on MRC-5 cell viability at any given concentration was tested at least as 4 replicates and were plotted as bar graphs based on average and standard deviation of these replicates.

### *M. tuberculosis* MIC assay

To exclude compounds with bactericidal or bacteriostatic properties, a *M. tuberculosis* MIC assay was performed in clear, round-bottomed 96-well plates (Brand^®^). Compounds were serially diluted in 7H9 medium with 0.1% Tween80 with a final volume of 100 μl in each well. Then, 100 μl of 0.02 OD_600_ *M. tuberculosis* H37Rv bacterial suspension was added into each well resulting in final compound concentration ranging from 2.5–40 μM. Isoniazid and rifampicin at varying concentrations were also used as additional controls. Each assay plate also had H37Rv without any compound as positive controls and medium-only negative controls. Plates were incubated at 37 °C standing for 7 days. Plates were then scored based on pellet formation in the bottom of the wells and spotted on 150 mm diameter 7H10 plates using a 96 solid pin multi-blot replicator (V&P Scientific). The 7H10 plates were incubated for 13 days at 37 °C and then colony formations on the plates were scored.

### Infection of macrophage-like cells

To measure the impact of our experimental compounds on intracellular survival, 2 × 10^4^ J774A.1 cells/well were seeded into to clear, flat bottomed 96 well plates (Thermo Scientific) and infected with *M. tuberculosis* H37Rv at a MOI of 10 bacteria/cell. After four hours, the wells were washed thrice with warm cell medium to remove extracellular bacteria and 100 µl of medium with 40 µM of either eburnamonine, CBK057809, CBK041705, CBK261420 or CBK261410 respectively. To control wells 0.4% DMSO or 0.24 μg/ml isoniazid was added. Plates were incubated at 37 °C with 5% CO_2_. At 0, 2 and 4 days cells were washed three times with warm medium and lysed by suspension in 0.06% SDS in 7H9 medium. The suspensions containing intracellular bacteria were serially diluted and plated onto 7H10 plates for viable count three weeks later. To test for cell toxicity in J774A.1 cells four days post infection, assay plates prepared as described above were washed three times with warm medium and incubated with 100 μl of 100 μM resazurin for another 4 hours at 37 °C. The plates were analysed by absorbance measurements at 595 nm and 570 nm on EMax plus plate reader as described above.

## Supplementary information

Supplementary information
