# Supplementary material for: Mycobacterium tuberculosis virulence inhibitors discovered by Mycobacterium marinum high-throughput screening
Source: Sci Rep. 2019 Jan 10;9:26. doi: 10.1038/s41598-018-37176-4 (PMC6328581; doi:10.1038/s41598-018-37176-4)
Supplement: Supplementary file 1 — Supplementary information [file 41598_2018_37176_MOESM1_ESM.pdf]

Supplementary information: *Mycobacterium tuberculosis* virulence inhibitors discovered by *Mycobacterium marinum* high-throughput screening

Hasan Tükenmez[1], Isabel Edström[2], Ramesh Ummanni[3], Stina Berglund Fick[4], Charlotta Sundin[3], Mikael Elofsson[3] and Christer Larsson[1]*.

[1] Infectious Diseases Clinic, Umeå University Hospital 901 85 Umeå, Sweden.

[2] Department of Molecular Biology, Umeå University 901 87 Umeå, Sweden.

[3] Department of Applied Biology, CSIR-Indian Institute of Chemical Technology (CSIR-IICT), Tarnaka, Hyderabad-500007, Telangana, India.

[4] Department of Chemistry, Umeå University 901 87 Umeå, Sweden.

*Correspondence to christer.larsson@umu.se

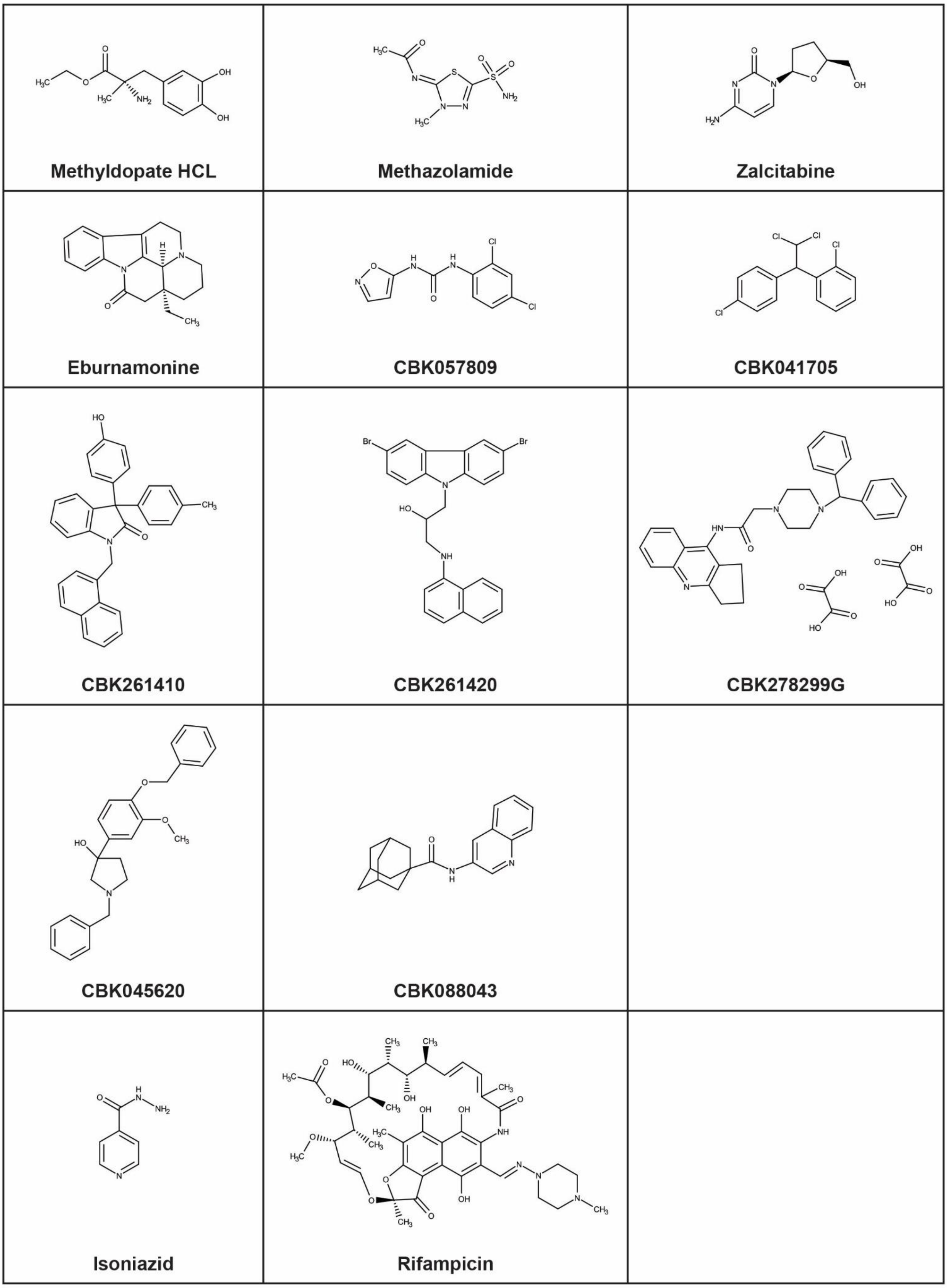

Supplementary Figure 1. Chemical structures of hit compounds selected for *M. tuberculosis* validation including first-line antibiotics isoniazid and rifampicin.

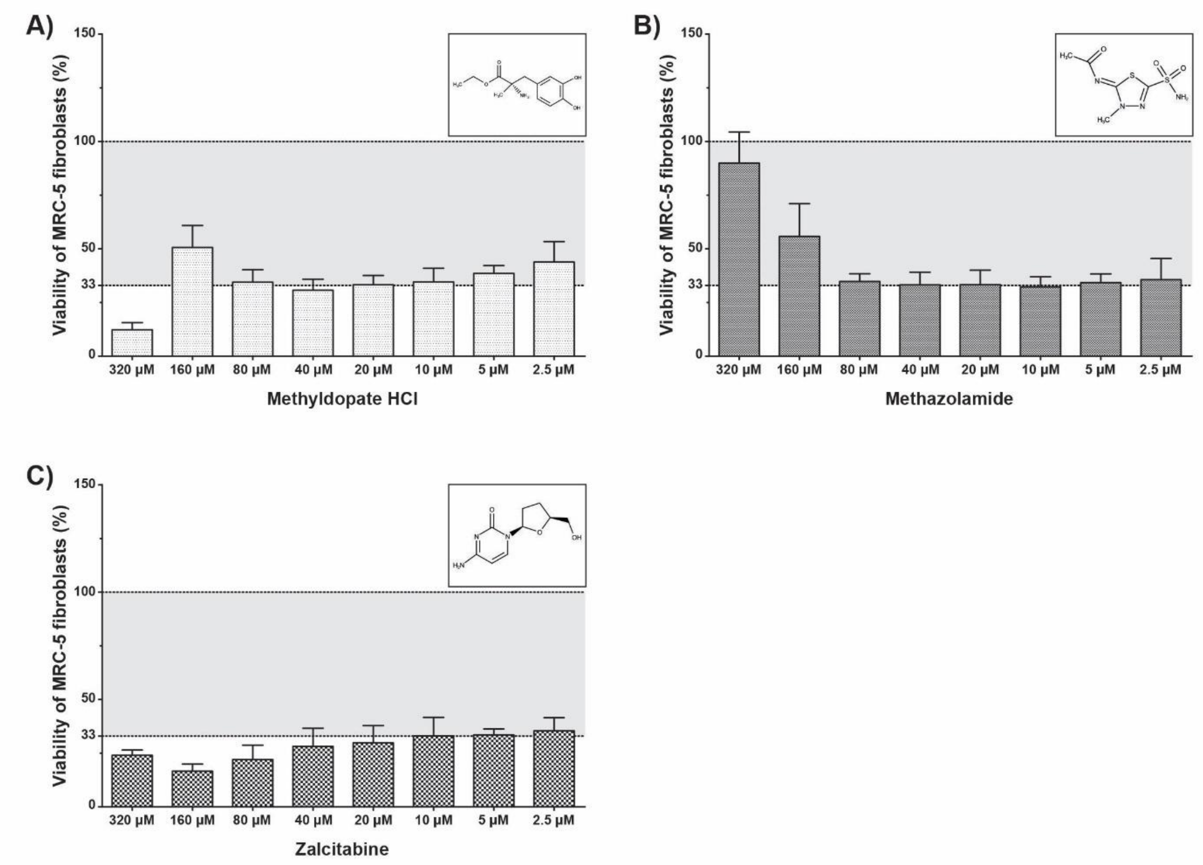

Supplementary Figure 2. Survival of MRC-5 fibroblasts after two days of infection with *M. tuberculosis* in the presence of various concentrations of (A) methyldopate HCl, (B) methazolamide or (C) zalcitabine. The viability of MRC-5 fibroblasts was determined by resazurin conversion assay (see materials and methods). The viability of MRC-5 fibroblasts that were exposed to avirulent *M. tuberculosis* H37Ra was set to 100% (upper dotted line) and the rest of the samples were normalized accordingly. In the absence of any compounds, exposure to virulent H37Rv results in 33% viability compared to H37Ra exposure (lower dotted line). The bar graph was plotted based on average and standard deviation values obtained from at least four independent replicates.

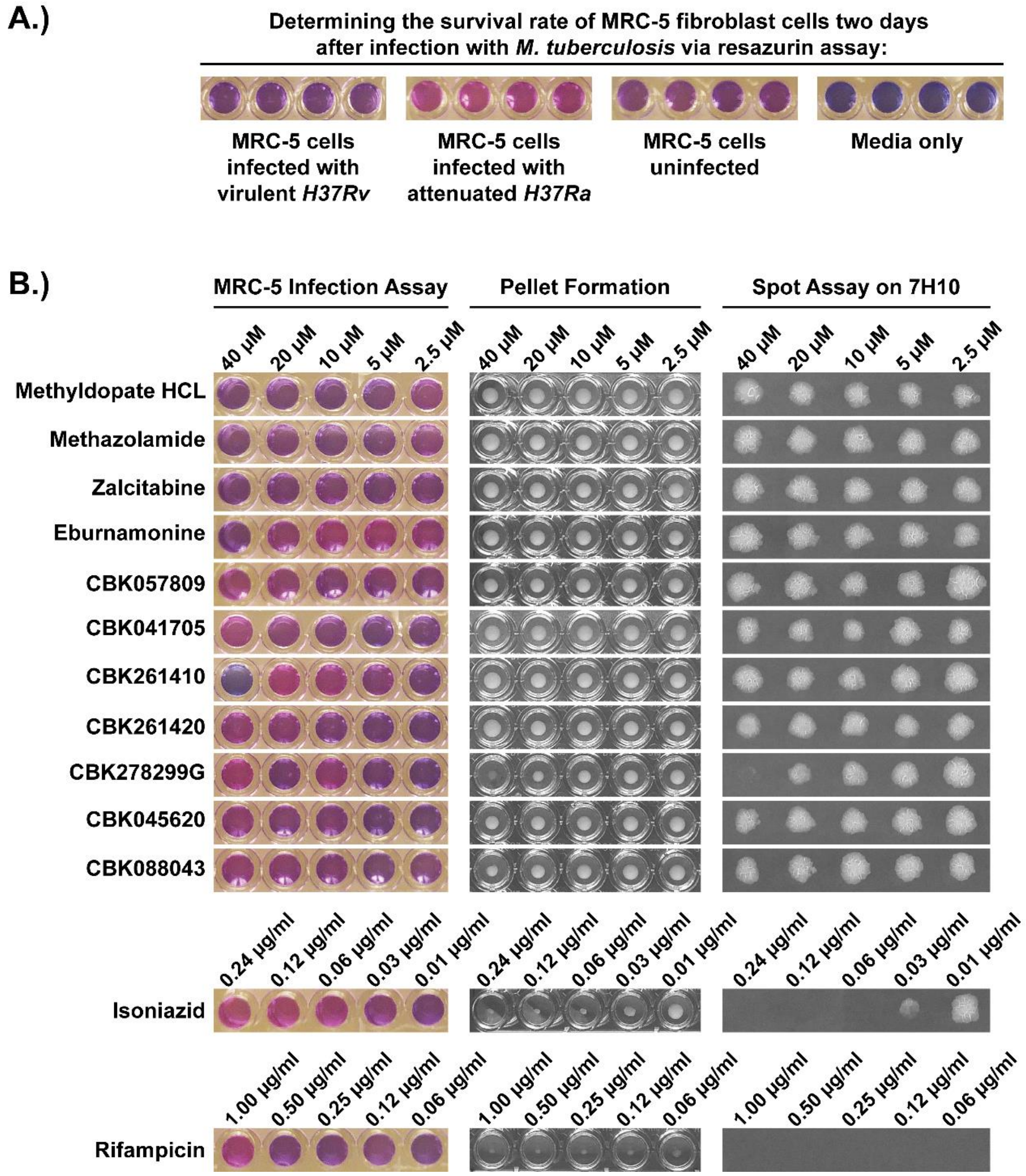

Supplementary Figure 3. Representative results from MRC-5 infection and antimicrobial assays with *M. tuberculosis* exposed to selected hit compounds. A) Resazurin conversion assay used to determine the survival of MRC-5 fibroblasts after two days of exposure to (from left to right) WT *M. tuberculosis* H37Rv, attenuated H37Ra, uninfected MRC-5 and media only. The figure shows quadruplicates from a representative experiment. **B)** Representative image from resazurin conversion assay showing dose response of the hit compounds including first-line antibiotics isoniazid and rifampicin in survival of MRC-5 fibroblasts upon infection with H37Rv (left). Representative images showing effect of the hit compounds on bacterial growth scored by pellet formation (center) and bacterial survival scored by spotting bacteria exposed to compounds on antibiotic-free 7H10 plates (right).

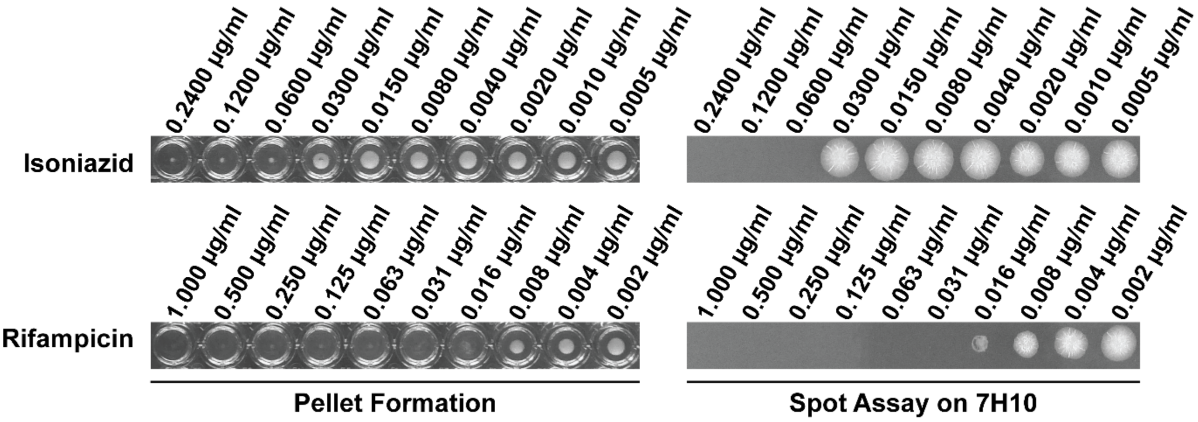

Supplementary Figure 4. Dose response of isoniazid and rifampicin. Representative images showing effect of isoniazid and rifampicin on bacterial growth scored by pellet formation (left) and bacterial survival scored by spotting bacteria exposed to compounds on antibiotic-free 7H10 plates (right).

Supplementary Table 1. Physicochemical properties* of hit compounds selected for *M. tuberculosis* validation including first-line antibiotics isoniazid and rifampicin.

Compounds	mol_MW	PSA	donorHB	accptHB	QPlogPo/w	QPlogS	QPPCaco	QPlogBB	QPPMDCK	#metab	Percent Human Oral Absorption	Rule Of Five	Rule Of Three
Methyldopate HCl	239.27	99	4.0	4.5	0.2	-1.1	50	-1.0	22	4	59	0	0
Methazolamide	236.26	118	2.0	8.5	-1.1	-1.4	85	-1.5	60	1	55	0	0
Zalcitabine	211.22	98	3.0	7.4	-0.5	-1.7	168	-1.2	72	2	64	0	0
Eburnamonine	294.40	34	0.0	5.0	2.4	-2.2	951	0.5	519	3	95	0	0
CBK057809	272.09	75	2.0	3.5	1.7	-3.2	395	-0.4	1501	0	84	0	0
CBK041705	320.05	0	0.0	0.0	6.6	-6.5	9906	0.2	10000	2	100	1	1
CBK261410	455.56	45	1.0	3.8	6.6	-7.5	1900	-0.3	990	3	100	1	1
CBK261420	524.25	32	2.0	2.7	7.1	-8.2	4789	0.2	10000	4	100	2	1
CBK278299G	476.62	53	1.0	7.5	4.9	-5.0	197	0.4	104	8	97	0	1
CBK045620	389.49	38	1.0	4.3	5.5	-5.5	1216	0.2	676	6	100	1	0
CBK088043	306.41	44	1.0	3.5	4.1	-5.2	3420	0.0	1869	1	100	0	0
Isoniazid	137.14	82	3.0	4.5	-0.6	-0.1	274	-0.8	122	2	67	0	0
Rifampicin	822.95	211	5.0	20.1	2.2	-1.3	6	-2.2	3	10	15	3	2

Property or Descriptor	Range for 95% of Known Drugs	Description
mol_MW	130.0 – 725.0	Molecular weight of the molecule
PSA	7.0 – 200.0	Van der Waals surface area of polar nitrogen and oxygen atoms
donorHB	0.0 – 6.0	Estimated number of hydrogen bonds that would be donated by the solute to water molecules in an aqueous solution. Values are averages taken over a number of configurations, so they can be non-integer.
accptHB	2.0 – 20.0	Estimated number of hydrogen bonds that would be accepted by the solute from water molecules in an aqueous solution. Values are averages taken over a number of configurations, so they can be non-integer.
QPlogPo/w	−2.0 – 6.5	Predicted octanol/water partition coefficient.
QPlogS	−6.5 – 0.5	Predicted aqueous solubility, logS. S in mol dm−3 is the concentration of the solute in a saturated solution that is in equilibrium with the crystalline solid.
QPPCaco	<25 is poor<br>>500 is great	Predicted apparent Caco-2 cell permeability in nm/sec. Caco-2 cells are a model for the gut-blood barrier. QikProp predictions are for non-active transport.
QPlogBB	−3.0 – 1.2	Predicted brain/blood partition coefficient. Note: QikProp predictions are for orally delivered drugs so, for example, dopamine and serotonin are CNS negative because they are too polar to cross the blood-brain barrier
QPPMDCK	<25 is poor<br>>500 is great	Predicted apparent MDCK cell permeability in nm/sec. MDCK cells are considered to be a good mimic for the blood-brain barrier. QikProp predictions are for non-active transport.
#metab	1 – 8	Number of likely metabolic reactions.
Percent Human Oral Absorption	<25% is poor<br>>80% is high	Predicted human oral absorption on 0 to 100% scale. The prediction is based on a quantitative multiple linear regression model. This property usually correlates well with HumanOralAbsorption, as both measure the same property.
Rule-Of-Five	maximum is 4	Number of violations of Lipinski's rule of five. The rules are: MW < 500, QPlogPo/w < 5, donorHB ≤ 5, accptHB ≤ 10. Compounds that satisfy these rules are considered drug-like. (The "five" refers to the limits, which are multiples of 5.)
Rule-Of-Three	maximum is 3	Number of violations of Jorgensen's rule of three. The three rules are: QPlogS > -5.7, QPPCaco > 22 nm/s, # Primary Metabolites < 7. Compounds with fewer (and preferably no) violations of these rules are more likely to be orally available.

* Physicochemical properties were calculated on low-energy 3D confirmation of the molecules in their neutral protonation state.
